# Preoperative mechanical bowel preparation with oral antibiotics reduces surgical site infection after elective colorectal surgery for malignancies: results of a propensity matching analysis

**DOI:** 10.1186/s12957-020-1804-4

**Published:** 2020-02-11

**Authors:** Purun Lei, Ying Ruan, Xiaofeng Yang, Juekun Wu, Yujie Hou, Hongbo Wei, Tufeng Chen

**Affiliations:** 1 Department of Gastrointestinal Surgery, The Third Affiliated Hospital, Sun Yat-sen University, Guangzhou, 0086-510000 China; 2 Department of Thyroid and Breast Surgery, The Third Affiliated Hospital, Sun Yat-sen University, Guangzhou, China; 3 Department of Gastrointestinal Surgery, The Sixth Affiliated Hospital, Sun Yat-sen University, Guangzhou, China

**Keywords:** Bowel preparation, Oral antibiotics, Surgical site infection

## Abstract

**Background:**

Surgical site infections (SSIs) are a major postoperative complication after colorectal surgery. Current study aims to evaluate prophylactic function of oral antibiotic (OA) intake in combination with mechanical bowel preparation (MBP) relative to MBP alone with respect to postoperative SSI incidence.

**Methods:**

A retrospective analysis of eligible patients was conducted using the databases of the Gastrointestinal Surgery Centre, Third Affiliated Hospital of Sun Yat-sen University from 2011 to 2017. Data pertaining to postoperative hospital stay length, expenses, SSI incidence, anastomotic fistula incidence, and rates of other complications were extracted and compared. A propensity analysis was conducted to minimize bias associated with demographic characteristics. Subgroup analyses were performed to further explore protective effects of OA in different surgical sites.

**Results:**

The combination of OAs and MBP was related to a significant decrease in the incidence of overall SSIs, superficial SSI, and hospitalization expenses. The MBP + OA modality was particularly beneficial for patients undergoing left-side colon or rectum resections, with clear prophylactic efficacy. The combination of MPB + OA did not exhibit significant prophylactic efficacy in patients undergoing right hemi-colon resection. Age, surgical duration, and application of OA were all independent factors associated with the occurrence of SSIs.

**Conclusion:**

These results suggest that the combination of OA + MBP should be recommended for patients undergoing elective colorectal surgery, particularly for operations on the left side of the colon or rectum.

**Trial registration:**

NCT04258098. Retrospectively registered

## Background

Surgical site infections (SSIs) are a major postoperative complication after abdominal surgery, especially in the colorectal field [1]. With a reported incidence of over 20%, SSIs significantly increase the length of stay (LOS), readmission rate, expenses, and mortality [2, 3]. Therefore, the identification of an effective method of reducing SSI incidence is critically important [4]. Colonic bacterial florae are considered to be the major cause of SSIs after elective colorectal procedures, but the most effective means of decreasing this bacterial load remains under debate [5]. Preoperative mechanical bowel preparation (MBP) was first utilized by surgeons, as it can theoretically remove stool content and associated bacterial load within the bowel and surgical field, thus reducing the risk of SSIs [6]. More recently, as antibiotics have come to be widely utilized, the preoperative administration of unabsorbed oral antibiotics (OAs) in combination with MBP was widely conducted [2, 7].

Multiple trials have been performed to explore the best bowel preparation strategies, but their results remain controversial [8–10]. Since 2005, several RCTs and meta-analyses have demonstrated MBP alone was not associated with a reduced incidence of SSIs related to patients that did not undergo MBP, whereas MBP patients exhibited paradoxical increases in postoperative ileus, anastomotic leakage, and other complications [11–14]. Recently, the merit of OA and MBP has been rediscovered in several related retrospective studies, which demonstrated a significant decrease in the rate of SSIs [15–17]. However, as information in these trials were exacted from national databases without any detailed matching between patient groups, the existence of bias in these trials may affect the validity of their results. Furthermore, none of these studies assessed the relative prophylactic effects of the novel MBP mode in right or left-side colorectal surgery. Herein, we report on our experiences in a single-center comparison of MBP + OA with MBP alone, assessing the rates of prophylactic combinations between groups via propensity score matching and stratification.

## Method

### Study population

This retrospective study was approved by the Ethics committee of the Third Affiliated Hospital of Sun Yat-sen University. Eligible patients were identified by searching the database of the Gastrointestinal Surgery Centre, Third Affiliated Hospital of Sun Yat-sen University from 2011 to 2017. Patient inclusion criteria were as follows: (1) patient underwent elective colorectal resection to treat a malignancy, (2) patient baseline characteristics and operative information were available, and (3) MBP was performed before surgery, with or without OA.

Patient exclusion criteria were as follows: (1) emergency surgery; (2) MBP was not conducted due to ileus or patient refusal; (3) enough data was not available; (4) colorectal resection was performed due to benign disease; (5) the procedure was accompanied by other procedures that had the potential to contaminate the incision, such as cholecystectomy or appendectomy; and (6) patients underwent neoadjuvant radiotherapy before surgery.

The primary and secondary aims of the study have been stated in the latest manuscript. The primary aim was to evaluate prophylactic function of preoperative OA combined with MBP vs MBP alone in postoperative SSI incidence. The secondary aim was to explore the potential benefit on length of hospital stays of OA + MBP mode compared with simple MBP.

Application of preoperative antibiotics was under surgeons’ decisions; no patients withdrew during the study period. Either polyethylene glycol or magnesium sulfate was adopted as a laxative 1 day before surgery. Clyster was conducted on surgery morning. Streptomycin 1 g plus metronidazole 0.2 g was prescribed 3 times a day for 3 days before surgery in the OA + MBP group patients.

Intravenous antibiotic prophylaxis was based on local guidelines and resistance profiles: most of the patients received cefmetazole 2 g intravenous drip 30 min before incision and once every 12 h until 48 h after surgery. Patients with penicillin or cephalosporin allergy were given clindamycin 0.6 g twice a day. If the surgical procedure lasted more than 180 min, a booster dose of antibiotic was administered.

### Outcomes

Based on the preparation procedures employed, patients were divided into a mechanical preparation plus oral antibiotics group (MBP + OA group) and a simple MBP group. The following demographic, clinical, and pathological information were extracted from the database: age, gender, BMI, comorbidities, American Society of Anesthesiologists (ASA) score, operative duration, laparoscopic or laparotomy approach, surgical site, neoadjuvant chemotherapy, combination with multi-organ resection, TNM stage, and preoperative serum albumin level. Outcomes of interest were length of hospital stay (LOS), expense, and rates of postoperative complications, which included anastomotic leakage, SSIs, postoperative ileus, respiratory/urinary infection, deep vein thrombosis (DVT), and postoperative *Clostridium difficile* infection (CDI).

### Statistical analysis

Frequencies were presented for categorical variables, and means ± standard deviation were given for continuous variables. Pearson’s *χ*^2^ or Fisher’s exact tests were used to analyze categorical variables. Student’s *t* tests were used for analyzing normally distributed data; otherwise, Mann-Whitney *U* tests were used for continuous variables. Propensity score matching was performed for minimizing confounding based on TNM stage, laparoscopic or laparotomy approach, ASA score, gender, BMI, and neoadjuvant chemotherapy. A multivariate logistic regression model was used to identify independent SSI risk factors, and a stepwise forward method was used for variable selection (inclusion *p* < 0.05; permanence *p* < 0.1). The fit for this logistic regression was tested with the Hosmer and Lemeshow test. All data analyses were performed with SPSS v22 (Armonk, NY: IBM Corp).

## Result

### Unmatched patient characteristics

We analyzed retrospective data from a total of 806 patients between 2011 and 2017, using the database of the Third Affiliated Hospital of Sun Yat-Sen University. Of these patients, 581 met the inclusion criteria and were enrolled in our study (255 in the OA + MBP group and 326 in the MBP group). The average age was 59.78 ± 12.11 in the OA + MBP group and 59.22 ± 12.16 in the MBP group. The average BMI in these groups was 22.40 ± 3.59 and 22.93 ± 3.40, respectively. Male patients occupied 56.86% and 61.04% in each group. Colon cancer accounted for 94.12% and 72.70% of the patients in each group, with the majority of patients having either stage II or III disease, and most patients underwent laparoscopic procedures. However, the stage, tumor location, serum albumin, surgical approach, neoadjuvant chemotherapy history, and rectal resection proportion were significantly different between the groups. All these baseline characteristics are shown in Table 1.

**Table 1:** Characteristics and incidence of postoperative complications for OA + MBP and MBP groups

	OA + MBP	MBP	*p* value
Number	255	326	
Age	59.78 ± 12.11	59.22 ± 12.16	0.583
Gender (male/female)	145/110	199/127	0.309
BMI	22.40 ± 3.59	22.93 ± 3.40	0.066
Approach (open/lap)	46/209	25/301	< 0.05
Surgical time (min)	199.59 ± 70.50	205.55 ± 60.46	0.274
ASA (1/2/3/4)	145/93/17/0	178/107/41/0	0.053
Stage (1/2/3/4)	34/101/67/53	45/135/108/38	0.019
Neoadjuvant chemotherapy	7	46	< 0.05
Colon/rectum	240/15	237/89	< 0.05
Albumin	39.13 ± 4.28	40.33 ± 5.49	0.04
Postoperative LOS (day)	10.10 ± 5.19	9.20 ± 5.01	0.03
Expenses (kRMB)	56.74 ± 16.60	66.73 ± 25.66	< 0.05
Anastomotic fistula	3	10	0.126
SSIs	27 (100%)	54 (100%)	0.03
Superficial	12 (44.4%)	26 (48.1%)	0.114
Deep	3 (11.1%)	9 (16.7%)	0.182
Space	12 (44.4%)	19 (35.2%)	0.55
Ileus	16	10	0.06
Urinary infection	3	1	0.324
CDI	8	10	0.962
Pulmonary infection	17	11	0.066
Post-hemorrhage	1	1	1
DVT	0	2	0.507
Readmission	6	11	0.621

*BMI* body mass index, *ASA* American Society of Anesthesiologists grading, *LOS* length of stay, *CDI Clostridium difficile* infection, *SSIs* surgical site infections *DVT* deep vein embolism

### Unmatched case results

Postoperative complications within 30 days were reviewed using the patient database. Postoperative LOS was significantly longer in the OA + MBP group (10.10 ± 5.19 vs 9.20 ± 5.01 days; *p* = 0.03), while the expenses were significantly greater in the MBP group (56.74 ± 16.60 vs 66.73 ± 25.66 kRMB; *p* < 0.05). The overall SSI incidence was significantly lower in the OA + MBP group (27 vs 54, *p* = 0.03 s), while rates of other complications such as anastomotic leakage, ileus, pulmonary infection, diarrhea, DVT, and hemorrhage were comparable between both groups, as shown in Table 1.

### Characteristics of the propensity score-matched samples

As retrospective data, bias inherent in these patient groups may influence study outcomes. To better control confounding variables and achieve comparable distributions of TNM stage, surgical approach, ASA score, gender, BMI, tumor location, and neoadjuvant chemotherapy history, patients were matched 1:1 based on those factors using SPSS. The propensity score-matched sample was comprised of 428 patients (214 in each group). After matching, all variants were similar between both groups as shown in Table 2.

**Table 2:** Characteristics and incidence of postoperative complications for propensity matched OA + MBP and MBP groups

	OA + MBP	MBP	*p* value
Number	214	214	
Age	59.37 ± 11.76	59.36 ± 12.11	0.994
Gender (male/female)	120/94	118/96	0.931
BMI	22.66 ± 3.63	22.42 ± 3.29	0.468
Approach (open/lap)	18/196	22/192	0.691
Surgical time (min)	196.79 ± 68.72	202.06 ± 55.63	0.384
ASA (1/2/3/4)	113/85/16/0	127/68/19/0	0.227
Stage (1/2/3/4)	31/91/57/35	22/93/76/23	0.08
Neoadjuvant chemotherapy	7	7	1
Colon/rectum	200/14	190/24	0.089
Albumin	39.43 ± 4.23	40.15 ± 5.26	0.124
Postoperative LOS (day)	9.71 ± 4.93	9.08 ± 4.97	0.192
Expenses (kRMB)	56.98 ± 16.58	65.27 ± 20.13	0.000
Anastomotic fistula	2	4	0.685
SSIs	15 (100%)	34 (100%)	0.004
Superficial	5 (33.3%)	15 (44.1%)	0.03
Deep	2 (13.3%)	5 (14.7%)	0.449
Space	8 (53.3%)	14 (41.2%)	0.189
Ileus	10	7	0.458
Urinary infection	2	1	1
CDI	7	8	0.793
Pulmonary infection	12	5	0.135
Post-hemorrhage	1	0	1
DVT	0	1	1
Readmission	4	3	1

*BMI* body mass index, *ASA* American Society of Anesthesiologists grading, *LOS* length of stay, *CDI Clostridium difficile* infection, *SSIs* surgical site infections, *DVT* deep vein embolism

### Results of the propensity score-matched sample

The matched data exhibited consistent results with respect to SSI incidence, with 15 and 35 cases in the OA + MBP and MBP alone groups, respectively (*p* < 0.05). There was also a significant difference in the rates of superficial SSI (5 vs 15 cases; *p* = 0.03), and total expense remained significantly different between groups (56.98 ± 16.58 vs 65.27 ± 20.13 kRMB; *p* < 0.05). The postoperative LOS no longer remained significantly different between groups after adjustment (9.71 ± 4.93 vs 9.08 ± 4.97 days, *p* = 0.192). Other outcomes remained comparable, as shown in Table 2.

### Subgroup analysis

To further explore the site-specific benefits of OA, all patients were subdivided into right hemi-colon and left-side colon or rectum subgroups, while patients that underwent transverse colectomies were excluded, after which propensity matching was performed. A total of 114 patients were included in the right hemi-colon subgroup. Patient baseline characteristics were comparable between the two groups. All postoperative outcomes were similar except expense (58.46 ± 21.29 vs 66.15 ± 14.44 kRMB; *p* = 0.03). Characteristics and results are shown in Table 3.

**Table 3:** Characteristics and postoperative complications incidence of the propensity matched data in right hemicolectomy subgroup

	OA + MBP	MBP	*p* value
Number	57	57	
Age	57.93 ± 11.43	59.28 ± 12.19	0.543
Gender (male/female)	30/27	33/24	0.572
BMI	22.88 ± 3.16	21.96 ± 3.35	0.135
Approach (open/lap)	8/49	9/48	0.793
Surgical time (min)	216.65 ± 82.23	207.71 ± 46.47	0.477
ASA (1/2/3/4)	26/24/7	25/25/7	0.980
Stage (1/2/3/4)	7/24/16/10	3/28/20/6	0.340
Neoadjuvant chemotherapy	1	1	1
Albumin	39.10 ± 4.40	38.29 ± 5.559	0.397
Postoperative LOS (day)	10.09 ± 6.91	9.51 ± 3.79	0.58
Expenses (kRMB)	58.46 ± 21.29	66.15 ± 14.44	0.03
Anastomotic fistula	1	0	1
SSIs	7 (100%)	9 (100%)	0.590
Superficial	1 (14.3%)	3 (33.3%)	0.618 s
Deep	1 (14.3%)	2 (22.2%)	1
Space	5 (71.4%)	4 (44.4%)	1
Ileus	3	0	0.243
Urinary infection	1	0	1
CDI	0	2	0.496
Pulmonary infection	6	3	0.490
Readmission	1	0	1

*BMI* body mass index, *ASA* American Society of Anesthesiologists grading, *LOS* length of stay, *CDI Clostridium difficile* infection, *SSIs* surgical site infections, *DVT* deep vein embolism

Left-side colon or rectum subgroup included left colectomy, sigmoid colectomy, and rectal resection patients. The SSI incidence and superficial SSI incidence were statistically different between both groups (13 vs 31, *p* = 0.004 and 7 vs 17, *p* = 0.032, respectively). The total expense was also higher in the MBP group (54.25 ± 14.25 vs 67.67 ± 34.68, *p* < 0.05), as shown in Table 4.

**Table 4:** Characteristics and postoperative complications incidence of the propensity matched data in left-side colon or rectum subgroup

	OA + MBP	MBP	*p* value
Number	124	124	
Age	60.81 ± 12.00	60.04 ± 11.25	0.605
Gender (male/female)	77/47	66/58	0.157
BMI	22.95 ± 3.35	23.42 ± 3.40	0.268
Approach (open/lap)	6/118	12/112	0.142
Surgical time (min)	189.67 ± 63.71	199.86 ± 60.77	0.200
ASA (1/2/3/4)	68/48/8	69/37/18	0.071
Stage (1/2/3/4)	22/47/34/21	19/48/38/19	0.907
Neoadjuvant chemotherapy	4	6	0.749
Albumin	40.07 ± 3.88	40.99 ± 5.29	0.12
Postoperative LOS (day)	9.79 ± 4.68	9.89 ± 6.21	0.881
Expenses (kRMB)	54.25 ± 14.25	67.67 ± 34.68	< 0.05
Anastomotic fistula	2	5	0.446
SSIs	13 (100%)	31 (100%)	0.004
Superficial	7 (53.8%)	17 (54.9%)	0.032
Deep	1 (7.7%)	5 (16.1%)	0.213
Space	5 (38.5%)	9 (29.0%)	0.271
Ileus	8	6	0.582
Urinary infection	2	1	1
CDI	5	3	0.722
Pulmonary infection	3	2	1
Readmission	3	1	0.622

*BMI* body mass index, *ASA* American Society of Anesthesiologists grading, *LOS* length of stay, *CDI* C*lostridium difficile* infection, *SSIs* surgical site infections, *DVT* deep vein embolism

To further analyze risk factors affecting SSI incidence, multivariate logistic regression was performed to evaluate the following parameters: ASA stage, age > 60, gender, surgical approach (laparoscopic approach), surgical time > 4 h, stage over II, application of preoperative OA, and low albumin levels. We found that age > 60 and surgical time > 4 h were independent risk factors for SSIs, whereas application of preoperative OAs was a protective factor reducing the incidence of SSIs as shown in Table 5.

**Table 5:** Logistic regression model to identify factors independently associated with surgical site infections

	SE	*B*	*p* value	OR	95% CI	
Age > 60	0.309	0.739	0.017	2.093	1.143	3.833
Time > 4 h	0.308	0.993	0.001	2.698	1.472	4.944
OA + MBP	0.336	− 1.007	0.002	0.365	0.192	0.693

*OA* oral antibiotics, *MBP* mechanical bowel preparation, *SE* standard error, *OR* odds ratio, *CI* confidence interval

## Discussion

Surgical site infection is one of the most common complications after colorectal surgery, substantially increasing patient morbidity and expenses [1–3]. With the large burden of bacteria in the bowel, elective colorectal resections are associated with particularly high rates of SSIs [2, 4]. Bowel preparation modes prior to elective colorectal surgery have been varied for decades and aim to reduce the SSIs [8–14, 18].

MBP was initially performed preoperatively with the goal of reducing bacterial burden and human fecal content and to thereby decrease SSI rates [6]. However, as mentioned above, subsequent research demonstrated that MBP alone failed to achieve this objective [2, 6], instead causing paradoxical complications [11–14]. It has been proposed that when implemented in concert with OA administration, the MBP-mediated reduction in bacterial burden may guarantee better OA delivery to the entire length of the colon, improving prophylactic activity [19–22]. Furthermore, with the advent of the ERAS era, surgeons have sought to minimize perioperative physiologic perturbations, leading to increasing concern regarding and abandonment of the use of MBP or OA. One such concern is that the combinational preparation may prolong preoperative hospital stays and expenses, in addition to causing increased patient discomfort and reduced compliance. As such, there is a need to determine whether the combination of MBP + OA yields better patient outcomes. Recently, the combination of OA and MBP has been evaluated in several retrospective studies which demonstrated a significant decrease in the rate of SSIs [15–17]. However, dietary structure, BMI, lifestyle, and colonic flora differ between people from Eastern and Western nations. Furthermore, no previous studies have evaluated the value of OA in a site-specific manner in the colon/rectum. As such, we performed a propensity matching retrospective study with subgroup analyses in order to further evaluate the prophylactic value of OA.

The current study revealed that the application of MBP + OA can significantly decrease the overall incidence of SSIs (10.59% vs 16.56%, *p* = 0.03) and expenses (56.74 ± 16.60 vs 66.73 ± 25.66 kRMB, *p* < 0.05) relative to MBP alone in patients undergoing elective colorectal resection. However, postoperative LOS was longer in the MBP + OA group (10.10 ± 5.19 days vs 9.20 ± 5.01 days, *p* = 0.03). The incidence of anastomotic fistula, postoperative ileus, urinary infection, *Clostridium difficile* infection, pulmonary infection, hemorrhage, DVT, and 30-day readmission was comparable in both groups. Owing to the retrospective nature of this analysis, several patient baseline characteristics were different between groups, including serum albumin, surgical approach, neoadjuvant chemotherapy history, and rectal resection proportion, potentially confounding our results. As such, a propensity score matching analysis was conducted to normalize patient groups according to TNM stage, surgical approach, ASA score, gender, BMI, tumor location, and neoadjuvant chemotherapy history with a 1:1 ratio. This led us to analyze a total of 428 patients in the final data analysis, which revealed that overall SSI incidence (7.01% vs 15.89%, *p* = 0.004), superficial SSI incidence (2.34% vs 7.01%, *p* = 0.03), and hospitalization expense (56.98 ± 16.58 vs 65.27 ± 20.13 kRMB, *p* < 0.05) were significantly higher in the MBP alone group. Postoperative LOS was comparable between both groups, potentially because patients with superficial SSI were first discharged and undergoing outpatient wound care, potentially influencing the LOS results in our center.

Previous studies have indicated that in the right hemi-colon, the concentration of bacteria ranges from 10^6^ to 10^7^ bacteria/g of stool content, whereas these numbers rise to 10^11^–10^12^ bacteria/g in the rectosigmoid region [6, 19]. Several studies have demonstrated a lower risk of SSIs for right colon resections [23, 24]. Therefore, the proposal to forgo the use of OA prior to right hemicolectomy was raised in the ERAS era, without formal demonstration of the outcomes of such an approach. Hence, in the present study, we performed for the first time a stratified propensity analysis of patient outcomes for right hemicolectomy and left-side (descending, sigmoid colon, and rectum) colorectal resection subgroups. The preventative function was especially prominent in left-side patients, in whom the incidence of overall SSIs (7.01% vs 15.89%, *p* = 0.004), superficial SSI (2.34% vs 7.01%, *p* = 0.03), and hospitalization expenses (56.98 ± 16.58 vs 65.27 ± 20.13 kRMB, *p* < 0.05) were significantly higher relative to the MBP alone group. However, similar improvements in outcomes upon OA administration were not observed in the right hemicolectomy subgroup. These findings are consistent with previous proposals, underscoring the need to tailor bowel preparation strategies based on the surgical site in a given patient.

Our logistic regression model demonstrated that SSI incidence was associated with age, surgical duration, and the application of OA. Together our findings demonstrate the key value of OA in bowel preparation for patients undergoing colorectal surgery, suggesting that individual preoperative evaluation may help avoid unnecessary bowel preparation and minimize postoperative morbidity.

There are several strengths to our study. Our detailed database provided us with a sufficient sample size to analyze the association between bowel preparation mode and postoperative outcomes. The propensity analysis helped minimize the bias in the baseline characteristics of enrolled patients and thus enhanced the generalizability of our findings. However, several limitations still exist in our trial. First, this was a single-institution study, which limits its external validity. Second, the bowel preparation modes were reviewed through the database and medical records, and as such, the compliance and quality could not be evaluated. Third, as in other retrospective studies, historical bias may still exist despite our propensity analysis.

With increasing adoption of ERAS, fewer patients will undergo bowel preparations. However, as this was a retrospective analysis, all reviewed patients were from an era prior to ERAS application. Therefore, comparisons between no bowel preparation, simple MBP, OA + MBP, and simple OA could not be conducted. We are now in the process of conducting a randomized controlled trial of the effects of MBP + OA compared with MBP alone (NCT03856671). With the application of ERAS, future assessments of patients who receive no MBP or simple OA will be conducted, thereby helping to overcome the limitations of the present study.

## Conclusion

The combination of oral antibiotics and mechanical bowel preparation was associated with a significant decrease in the overall incidence of SSIs, superficial SSI, and hospitalization expenses. MBP + OA is therefore recommended, especially in patients undergoing left-side colon or rectum resections given its clear prophylactic efficacy. The MPB + OA combination provided no clear benefit in right hemi-colon resection patients. Age, surgical duration, and application of OA were independent factors that affected the rate of SSI occurrence.

## Data Availability

The datasets used and analyzed during the current study are available from the corresponding author on reasonable request.
